# Diode and er: YAG laser therapy versus other desensitizing treatments for dentin hypersensitivity in molar-incisor hypomineralization: a systematic review

**DOI:** 10.1007/s10103-026-04830-7

**Published:** 2026-03-12

**Authors:** Eliane Cardoso Cappellaro, Jéssica Fogliato Ribeiro, Carolina Lopes da Silva, Cleber Paradzinski Cavalheiro, Manoela Domingues Martins, Tathiane Larissa Lenzi

**Affiliations:** Federal University of Rio Grande Do Sul, Porto Alegre, Brazil

**Keywords:** MIH, Dentin sensitivity, Lasers, Dentin hypersensitivity, Systematic review

## Abstract

This study investigated the use of laser therapy for dentin hypersensitivity (DH) in patients with molar-incisor hypomineralization (MIH), compared with other desensitizing treatments. A systematic search was performed in electronic databases: PubMed, Scopus, LILACS, Embase, and Web of Science. Additionally, trial registries (ClinicalTrials.gov and ReBEC), grey literature, and manual search were checked. Two reviewers independently performed study selection, data extraction, and risk of bias assessment using the RoB 2 tool. Given the heterogeneity in included studies, only a descriptive analysis was undertaken. From 2,450 potentially eligible studies, three randomized clinical trials were selected for full-text analysis, and included. These studies comprising 462 MIH-affected molars and incisors in patients aged 6–14 years. Follow-up ranged from 1 to 6 months. Interventions included diode lasers (808 nm, 980 nm) and a low-power Er: YAG protocol (0.4 W, 40 mJ, 10 Hz), compared with fluoride varnish, GLUMA desensitizer, or CPP-ACPF mousse. All studies reported DH reduction across groups; however, no statistically significant differences were observed between laser and other desensitizing treatments. Two studies were rated as high risk of bias, and one showed some concerns. Available evidence remains limited and heterogeneous due to few included studies and an overall fragile evidence base, indicating that laser therapy may reduce DH in MIH-affected teeth. Well-designed and standardized randomized clinical trials are still needed to strengthen the evidence base. (PROSPERO registration: CRD42024608626).

## Introduction

Molar incisor hypomineralization (MIH) is a developmental qualitative enamel defect of one to four permanent first molars, often with associated incisor involvement [1]. It is presented as demarcated enamel opacities of different colors, occasionally undergoing posteruptive breakdown [1]. Patients with MIH often report dentin hypersensitivity (DH), a sharp and brief pain in response to mechanical and thermal stimuli [2]. Sensitivity can occur even in opacities without structural damage, ranging from mild reactions to spontaneous DH [3]. The global prevalence of DH/toothache in MIH patients is reported at 45% [4]. The mechanisms involved in DH in MIH-affected teeth remain unclear. Increased enamel porosity may diminish thermal insulation and alter fluid dynamics, facilitating irritant penetration into dentinal tubules and contributing to subclinical pulp inflammation and neuronal sensitization [5–7]. This heightened sensitivity may also impair biofilm control and increase the risk of caries, with potential negative effects on oral health-related quality of life [8, 9]. Given these clinical consequences, DH management focuses on reducing pain by inhibiting nerve impulses or promoting dentinal tubule occlusion. Several therapeutic strategies have been proposed, including fluoride applications, arginine/calcium carbonate, casein phosphopeptide–amorphous calcium phosphate (CPP-ACP), casein phosphopeptide amorphous calcium phosphate fluoride (CPP-ACPF), desensitizing agents, and laser therapy [10]. Photobiomodulation (PBM), delivered through low-level lasers therapy (LLLT) or LEDs, produces therapeutic effects via a non-thermal mechanism that activates endogenous chromophores and triggers photophysical and photochemical cascades, ultimately promoting tissue repair and providing anti-inflammatory and analgesic benefits [11]. The high-power laser acts directly on the dental tissue, promoting obliteration and/or reduction of the diameter of the dentinal tubules. It can also reduce DH by leading to the depolarization of the pulp nerve fibers [12]. Evidence indicates that laser therapy can significantly reduce dentin hypersensitivity both in the short and long term in patients without MIH [13]. However, whether these benefits extend to individuals with MIH remains uncertain. Existing evidence [11, 13] primarily compared laser therapy with placebo or no treatment, while a range of other desensitizing modalities, such as fluoride varnish, GLUMA, and CPP-ACPF mousse, are currently available in clinical practice. Therefore, this systematic review aimed to critically evaluate the available evidence on laser therapy for dentin hypersensitivity in MIH-affected patients, comparing its effects with those of other desensitizing treatments.

## Material and methods 

This systematic review was developed according to the Cochrane Handbook [14], reported in compliance with the Preferred Reporting Items for Systematic Reviews and Meta-Analyses (PRISMA) 2020 statement [15], and registered in the PROSPERO database (CRD42024608626) prior to its initiation.

### Review question and eligibility criteria 

The following research question was formulated to address the literature and outline the search strategy: Does laser therapy reduce the dentin hypersensitivity (DH) of teeth with MIH compared to others desensitizing treatment? The population/problem, intervention, comparison, outcome of the study and study design were established according to the PICOS question. In this respect, the population consisted of patients with hypersensitivity in teeth with MIH, without age restriction. The intervention was the use of laser therapy and the comparison was the application of any other desensitizing treatment, without association with laser therapy. The outcome evaluated was the reduction of DH in patients with MIH. Clinical trials were included, regardless of whether they were randomized or non-randomized, as this information was not always available in titles or abstracts; randomization status was confirmed during full-text review and used as an exclusion criterion.

### Search strategy

A comprehensive literature search was performed in June 2025 using the MEDLINE via PubMed, Scopus, LILACS, EMBASE, and Web of Science databases to identify studies related to the research question. No restrictions on publication year or language were applied to minimize publication bias. The following search steps were performed: computer search of databases and contact with authors.

To reduce the publication bias, the ClinicalTrials.gov website and the Brazilian Registry of Clinical Trials (ReBEC) website were checked. Also, grey literature was assessd by Google Scholar for unpublished documents and a review of reference lists of all included studies was done. The PubMed/MEDLINE search strategy was developed first, using a combination of MeSH terms and keywords with Boolean operators (AND/OR), and subsequently adapted for the syntax and indexing of the other databases (Table 1). Results obtained from different databases were checked against each other to ensure the removal of duplicates manually.

**Table 1:** Search strategies used for all databases consulted

Database	Search strategy
PubMed/MEDLINE	((((((((((((Hypersensitivity[MeSH Terms]) OR (Hypersensitivity[Text Word])) OR (Sensitivity[MeSH Terms])) OR (Sensitivity[Text Word])) AND (Desensitizer*[Text Word])) AND (Remineralization[Text Word])) AND (Molar hypomineralization[MeSH Terms])) OR (Molar hypomineralization[Text Word])) OR (Hypomineralization*[Text Word])) OR (Molar-incisor hypomineralization[Text Word])) OR (Molar incisor hypomineralization[Text Word])) OR (MIH[Text Word])) OR (Hypomineralization, molar incisor[Text Word])
EMBASE	molar incisor hypomineralization AND sensitivity
LILACS	(molar incisor hypomineralization) OR (hypomineralization) AND (fluoride varnish) AND (laser) AND (hipersensitivity) OR (sensitivity) AND (db: (“LILACS”))
Scopus	(molar AND incisor AND hypomineralization) OR (hypomineralization) AND (fluoride varnish) AND (laser) AND (hipersensitivity) OR (sensitivity)
Web of Science	molar incisor hypomineralization (All Fields) or hypomineralization (All Fields) and laser (All Fields) and fluoride varnish (All Fields) and sensitivity (All Fields) or hypersensitivity (All Fields)
REBEC	Molar incisor hipomineralization AND sensitivity OR hipersensitivity
Clinical Trials	Hypomineralization Molar Incisor | Other terms: Sensitivity
Google Scholar	(molar incisor hypomineralization) OR (hypomineralization) AND (fluoride varnish) AND (laser) AND (hipersensitivity) OR (sensitivity)

### Eligibility criteria

A brief calibration exercise was conducted prior to screening to ensure consistent application of eligibility criteria. Firstly, titles and abstracts were reviewed independently by two authors (E.C.C. and J.F.R.) and selected for further review if they met the inclusion criteria. The calculation of inter-examiner agreement (Kappa = 0.97) indicated excellent agreement. Full-text versions of articles selected in the previous step were retrieved and reviewed independently by two authors (E.C.C. and J.F.R.) considering the exclusion criteria: (1) non-random allocation of subjects, (2) absence of similar follow-up for subjects of both groups evaluated in the same way, (3) no computable data for both groups, (4) and use of unvalidated or ad hoc instruments to measure dentin hypersensitivity. Both the title/abstract screening and full-text assessment were conducted manually. Disagreements were firstly resolved by discussion between the reviewers (E.C.C. and J.F.R.). If discrepancies remained, a third author (T.L.L.) was consulted.

### Data extraction

Prior to full data extraction, the standardized sheet was pilot-tested to ensure consistency. Two authors (E.C.C. and J.F.R.) independently extracted data collected the data using a standardized sheet in Microsoft Office Excel 2013 (Microsoft Corporation, Redmond, WA, USA). For each paper, the following data were systematically extracted: publication details (title, authors, year of publication and country, funding sources and conflict of interest), methodology (sample size, age of participants, number of teeth included, type of tooth (incisors and molars), treatment protocol - laser (type of laser, time, place of application, number of applications) and protocol of other desensitizing treatment protocols, number of operator(s), outcome (method used to assess the hypersensitivity and main results). Any discrepancies between the reviewers were resolved through discussion, and if consensus could not be reached, a third author (T.L.L.) was consulted.

### Assessment of risk of bias 

The reviewers (E.C.C. and J.F.R.) also independently and in duplicate assessed the risk of bias using the RoB2 tool [16]. The criteria were divided into five domains as follows: bias arising from the randomization process, bias due to deviations from intended interventions, bias due to missing outcome data, bias in measurement of the outcome, and bias in selection of the reported result. The evaluation of the studies was performed by rating each domain as low risk of bias, some concerns, or high risk of bias. Inter-rater reliability was ensured through independent assessments conducted by both reviewers, and any disagreements were resolved by consensus; if consensus could not be reached, a third author (T.L.L.) was consulted. Final study-level judgments were determined using the RoB2 overall algorithm.

### Data analysis

The included studies had a high heterogeneity regarding laser protocols, other desensitizing treatments, DH assessment methods, and follow-up periods. Due to this substantial clinical and methodological variability, a meta-analysis was not feasible or methodologically justified. Therefore, a qualitative analysis was conducted based on the collected data. Heterogeneity was characterized descriptively by examining both methodological and clinical differences across the studies to ensure a coherent and comprehensive interpretation of the evidence.

## Results

### Study selection 

**Fig. 1:**
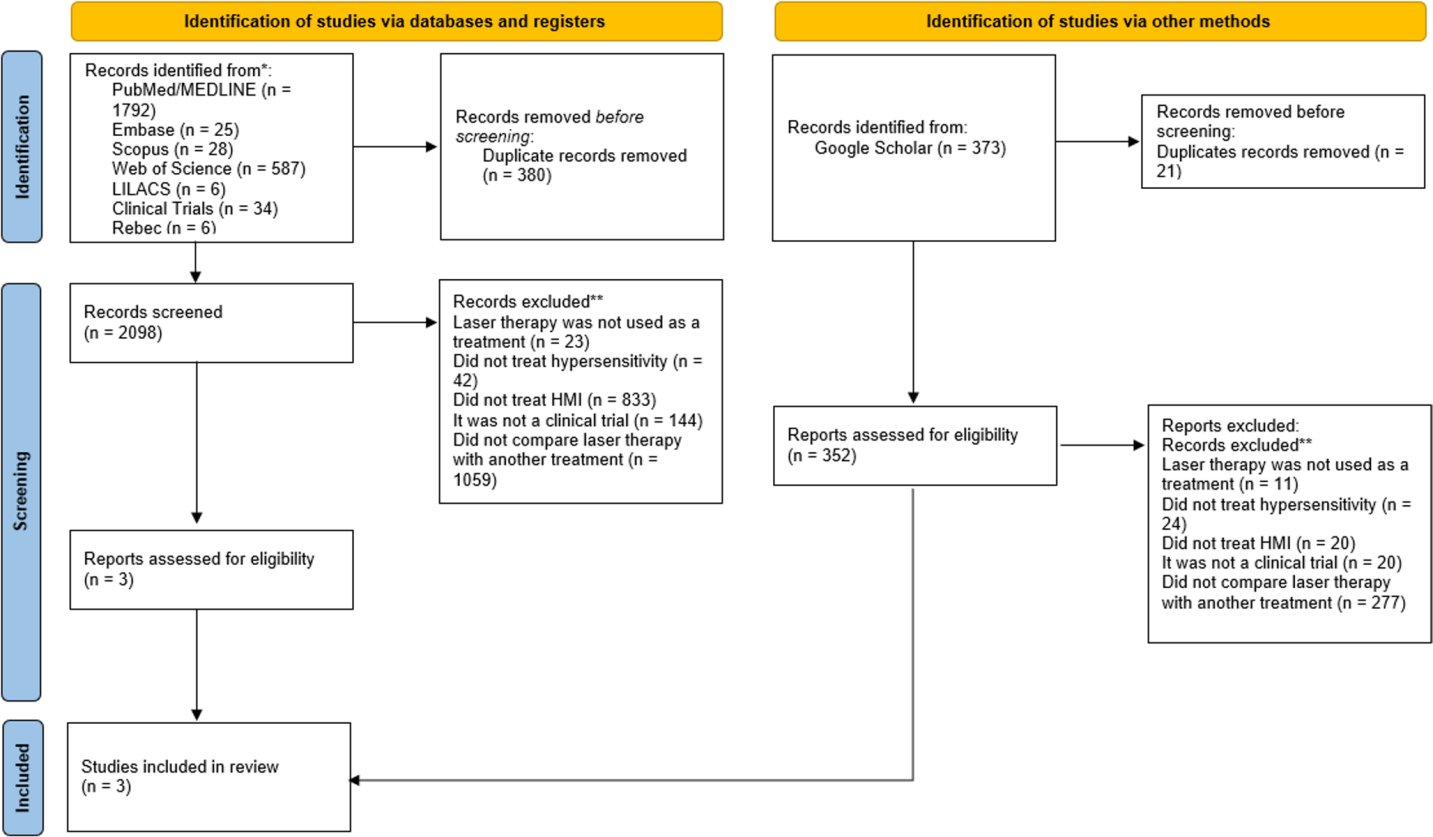
Flowchart of the study selection process and the reasons for exclusion

The search strategy identified 2,098 potentially relevant studies, excluding duplicates. After screening titles and abstracts, three studies were retrieved to obtain detailed information. Finally, these same three studies [17–19] met the eligibility criteria and were included in the qualitative analysis. Figure 1 summarizes the process of study selection and the reasons for exclusions.

### Assessment of risk of bias of the included studies

**Fig. 2:**
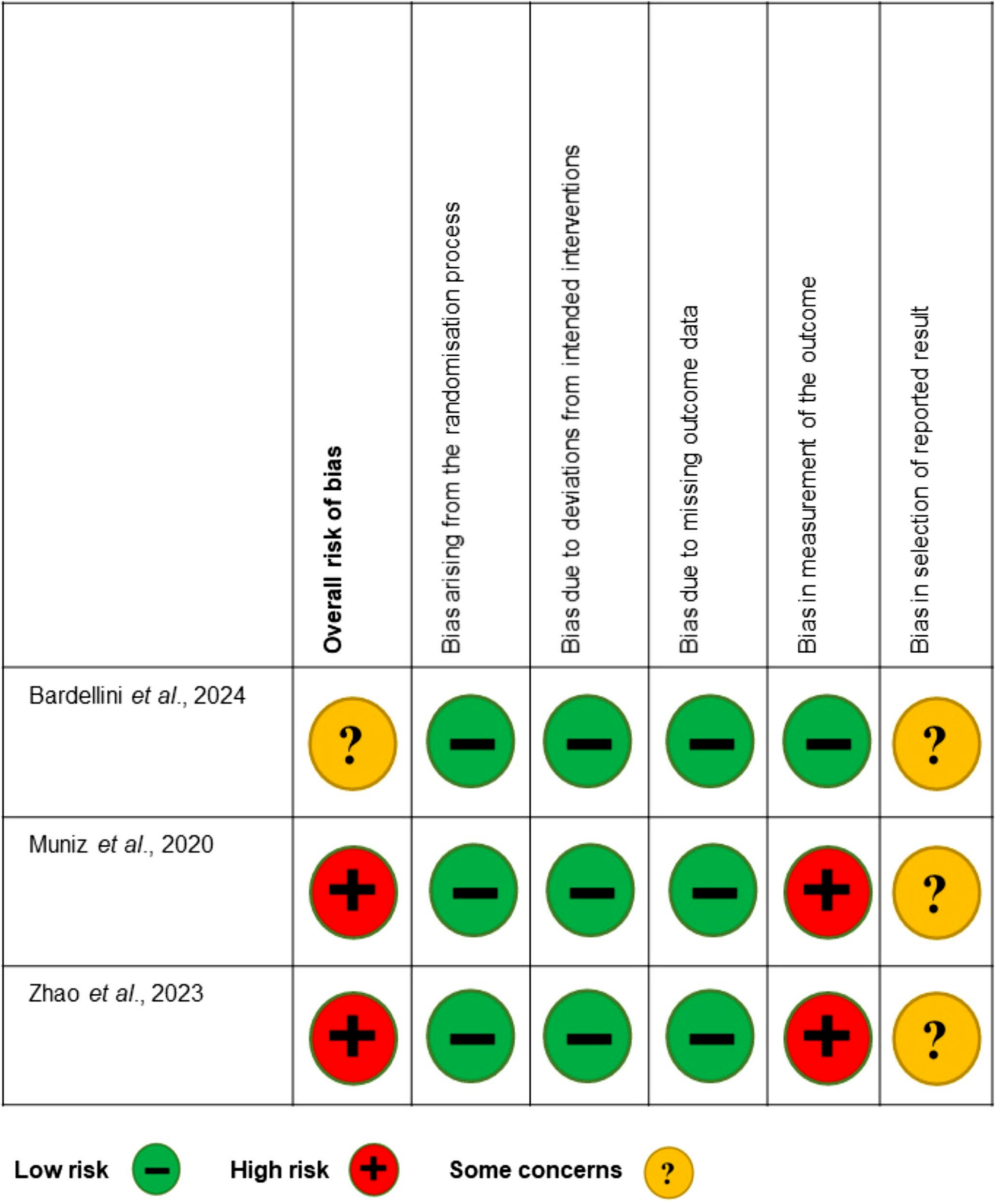
Risk of bias assessment of the included studies

Risk of bias assessment was performed and a detailed breakdown for each criterion is presented in Fig. 2. For the three selected studies, the overall risk of bias was judged as high for two studies [17, 18] and as some concerns for one study [19]. All the studies [17–19] were considered as low risk of bias regarding the randomization process, deviations from intended interventions and missing outcome data, indicating that study groups were comparable at baseline and that attrition or protocol deviations are unlikely to have substantially influenced the validity of the results. One study [19] was judged to be low risk of bias in measurement of the outcome as it specified that the evaluators who conducted the sensitivity assessment were blinded to the treatment groups; In the other two studies [17, 18], this information was not reported, resulting in a high risk of bias rating for this criterion. Given that DH is a patient-reported outcome, blinding of evaluators helps minimize potential bias in how the assessments were conducted or recorded. Bias in selection of reported result showed some concerns to all the three included studies [17–19].

### Characteristics of included studies

The main characteristics of the included studies are summarized in Table 2. Studies were conducted in Brazil [17], China [18], and Italy [19], and were published between 2020 and 2024. Collectively, they assessed 462 molars and incisors affected by MIH in a total of 131 participants aged 6 to 14 years, with the tooth serving as the unit of analysis. In all studies, MIH was diagnosed according to the European Academy of Paediatric Dentistry (EAPD) criteria [20]. DH was evaluated using the Visual Analogue Scale (VAS), sometimes in combination with the Wong-Baker Faces Pain Rating Scale (WBFPRS) [19], 14-item Oral Health Impact Profile (OHIP-14) and Likert Scale [18], and Pimenta Pain Intensity Face Scale [17]. Cold stimulus was used to evaluate the outcome.

**Table 2:** Main characteristics of datasets from selected studies from systematic review

	No. of participants and age in years	Teeth	Sensitivity assessment	Follow- Up	Drop- out		Source of funding	Conflict of interest
Muniz et al., 2020, [17] Brazil	44 (22 L and 22 FV). 6 to 12 years old.	157 (75 L, 82 FV).	Adapted Pimenta Pain Intensity Face Scale.	1 month.	4 (2 L, 2 FV).		Fundação de Amparo à Pesquisa e ao Desenvolvimento Científico e Tecnológico do Maranhão (FAPEMA)	Authors declare no conflict of interest.
Zhao et al., 2023, [18] China	56 (28 L and 28 DE). 11 to 14 years old.	199 (92 L, 107 DE).	VAS using OHIP-14.	1 week, 4 weeks and 6 months.	8 (4 L, 4 DE 4).	Six Talent Peaks Project in Jiangsu Province (2019 wsw-128) and “3456” Cultivation Program for Junior Talents of Nanjing Stomatological School, Medical School of Nanjing University (0222C114).		Authors declare no conflict of interest.
Bardellini et al., 2024, [19] Italy	27 (13 L and 14 CPP-ACPF). 6 to 14 years old.	106 (54 L, 52 CPP-ACPF).	VAS using WBFPRS.	7, 14 and 28 days.	0	This research received no external funding.		Authors declare no conflict of interest.

*L *lasertherapy, *FV* fluoride varnish, *DE* dessensitizing, *CCP-ACPF* casein phosphopeptide amorphous calcium phosphate fluoride, *VAS* Visual Analogue Scale, *OHIP* Oral Health Impact Profile, *WBFPRS* Wong-Baker Faces Pain Rating Scale

Two studies used low-level diode lasers: one with an 808 nm wavelength and 100 mW output power (Twin Flex Evolution, MM Optics, Brazil) [17], and another with a 980 nm wavelength, 4 W power, and an energy density of 60 J/cm² (RAFFAELLO 980 BIO, DMT S.r.l., Italy) [19]. The third study evaluated an Er: YAG laser at 2,940 nm (LiteTouch, Syneron/Light Instruments Ltd., Israel), which is typically a high-power system but was applied at low power (0.4 W, 40 mJ, 10 Hz) [18]. The control therapies involved the fluoride varnish applications (Duraphat, Colgate, USA) [17], the use of a desensitizer (GLUMA, Heraeus Kulzer, Germany) [18], and CPP-ACPF mousse (MI Paste Plus, GC, Italy) [19]. 

### Descriptive analysis

The treatments varied across studies in terms of laser type, intervention protocols, desensitizing control treatments, and follow-up duration. Table 3 provides a detailed summary of each included study, describing the intervention and comparator protocols, as well as the corresponding results. Across the three studies, all laser-based interventions resulted in an initial reduction in DH. However, the magnitude and durability of this effect differed depending on the laser parameters and the control treatment used. Muniz et al. (2020) [17] reported statistically significant DH reductions for both PBM (808 nm) and fluoride varnish, with PBM providing an immediate effect at 48 h, whereas fluoride varnish showed a delayed response. Both treatments achieved clinically meaningful reductions (79% and 87%, respectively), with no indication that these differences were statistically significant. Zhao et al. (2023) [18] also observed an immediate decrease in DH following Er: YAG laser application, whereas GLUMA demonstrated an early effect that was sustained up to 4 weeks. However, both treatments showed an increase in DH at 6 months, indicating limited long-term durability. Although the percentage reductions at six months were 50% (laser) and 28.6% (GLUMA), the difference was not statistically significant. Bardellini et al. (2024) [19] found no significant differences between diode laser (980 nm) and CPP-ACPF mousse in molars, regardless of the evaluation period. However, a greater reduction in DH was observed in incisors treated with laser therapy, suggesting a potential site-specific effect. Taken together, the studies indicate that laser therapy consistently produces an immediate reduction in DH across different wavelengths and protocols. However, sustained benefit varies substantially: one study reported continued improvement at 4 weeks [17], another reported rebound sensitivity by 6 months [18], and one found no significant differences between interventions over time for molars [19]. These patterns suggest that, although laser therapy appears to provide short-term improvements in DH, the extent to which these effects are maintained over the longer term remains unclear and may vary according to factors such as laser type and the control treatment.

**Table 3:** Summary of the included studies, detailing the intervention and comparator protocols and the corresponding results

	Laser protocol							Other desensitizing treatments			Main results
	Model and commercial brand	Wavelength	Power	Fluence (J/cm²)	Pulse duration	Total exposure time	Frequency of treatment	Desensitizing and commercial brand	Application protocol	Frequency of treatment	
Muniz et al., 2020 [17]	Low-level diode laser (Twin Flex Evolution, MM Optics, Brazil)	808 nm (infrared)	100 mW	NR.	NR	10 s (1 J) per point	2 sessions with a 48-hour interval	Varnish fluoride (Duraphat, Colgate, USA) – 22,600 ppm	Applications using microbrush to the entire lesion	One session per week for a period of one month	There was no significant difference in the mean VAS scores between the FV and L groups after the treatment.
Zhao et al., 2023 [18]	Er: YAG laser (LiteTouch, Syneron, Light Instruments Ltd., Israel)	2940 nm	0.4 W, 40 mJ, 10 Hz as a low-level erbium laser	NR	NR	20 s	A single session with three times of irradiation in 0.5 cm away from the tooth surface and kept moving for 20 s	GLUMA desensitizer (Heraeus Kulzer GmbH, Hanau, Germany)	Aapplication with a microbrush at the lesion for 60 s, dried with compressed air for 60 s and rinsed the tooth surface with water.	A single application	There was no significant difference in the mean VAS scores between the De and L groups after the treatment (*p* = 0.718) and in months (*p* = 0.524)
Bardellini et al., 2024 [19]	RAFFAELLO 980 BIO (Dental Medical Technologies — DMT S.r.l., Milan, Italy)	980 nm	4 W	60 J/cm²	NR	15 s per 1 cm²	2 sessions with a 7-day interval	CPP-ACPF mousse (MI Paste Plus, GC Italy Srl, Milan)	Application using a microbrush to the buccal cervical surface of each tooth for 5 min, distribute for 20 s using a rubber cup attached to a low-speed handpiece	Three sessions with a 7-day interval	CPP-ACPF mousse and PMBT demonstrated desensitizing effects.

*NR* not reported,* J* joule, *L* laser therapy, *FV *fluoride varnish,* DE* desensitizer, *CCP-ACPF* casein phosphopeptide amorphous calcium phosphate fluoride, *Er: YAG* Erbium-doped Yttrium Aluminum Garnet

## Discussion 

This systematic review examined the use of laser therapy for DH in MIH-affected teeth in comparison with other desensitizing treatments such as fluoride varnish, GLUMA, and CPP-ACPF mousse. We planned a priori to conduct a meta-analysis; however, it was not performed due to the substantial heterogeneity in study designs, laser parameters, intervention and comparision protocols, and follow-up durations. Consequently, a qualitative analysis was conducted to synthesize and interpret the findings across studies. In the included studies [17–19], laser therapy produced an immediate reduction in DH, although the duration of this effect varied depending on the type of laser, wavelength, and comparator treatment. Overall, no significant difference in DH reduction was observed among the treatments. In this systematic review, heterogeneity was characterized descriptively by examining both methodological and clinical differences across the included studies to ensure a coherent interpretation of the evidence. The three studies [17–19] employed distinct laser modalities: two used diode lasers with wavelengths of 808 nm [17] and 980 nm [19], respectively, and one used an Er: YAG laser with a wavelength of 2940 nm [18] operated at 0.4 W, 40 mJ, and 10 Hz as PBM. Substantial variation existed in power settings, irradiation techniques, number of sessions, and treatment application sites. These factors, combined with differences in the comparator treatments, contributed to pronounced clinical and methodological heterogeneity. Such variability not only prevented the execution of a meta-analysis but also limits the comparability of outcomes and reduces confidence in synthesizing unified conclusions regarding the effects of laser therapy for DH in MIH-affected teeth. PBM reduces the chronic cellular inflammation, activates Na⁺/K⁺ pumps, and stimulates tertiary dentin formation, promoting dentinal tubule obliteration [21]. It also provides analgesic, anti-inflammatory, and biomodulatory effects [12, 22], and its use for dentin hypersensitivity (DH) has been widely investigated beyond MIH-affected teeth. Studies indicate that PBM modulates neural responses and stimulates reparative mechanisms in pulp tissue [23, 24], partly due to its action on dental pulp stem cells, enhancing their activation, proliferation, and differentiation [23, 25]. These biomodulatory effects contribute not only to reducing hypersensitivity but also to promoting tissue regeneration through growth factor release and neural activity modulation [24, 25]. Regarding erbium lasers, Er: YAG is unlikely to cause thermal damage due to its high water absorption, which helps protect the pulp and dentin [26]. Its pulsed emission aids in dissipating heat during irradiation and reduces thermal influence on the pulp. The temperature increase generated by Er: YAG laser application promotes melting and recrystallization of dentin, resulting in dentinal tubule occlusion; a mechanism that supports its effectiveness in managing DH. Despite the biological plausibility and immediate clinical effects observed with laser therapy, the comparative effectiveness between laser and other standalone desensitizing strategies remained unclear. Existing evidence on MIH-associated DH has primarily compared laser therapy with placebo or no treatment [11, 13], or has evaluated protocols in which laser therapy was combined with other desensitizing strategies. A recent systematic review [27], reported that the association of LLLT with combined treatments may enhance long-term DH reduction, while LLLT alone provides an immediate effect on MIH-affected teeth. However, these studies did not isolate the performance of desensitizing agents used without laser application. Given this evidence gap, the present systematic review specifically compared laser therapy with desensitizing treatments applied independently, without any combination or adjunctive use of laser. Desensitizing agents reduce pain by either forming a protective coating over dentinal tubules or modifying their contents through mechanisms such as protein precipitation, coagulation, or the formation of insoluble calcium complexes [28]. Fluoride varnish creates a mechanical barrier and promotes dentinal tubule occlusion [29], hereby minimizing the penetration of external stimuli into dentin and reducing the onset of DH. CPP is a casein-derived phosphopeptide capable of binding and stabilizing soluble ACP. Upon application, ACP dissociates into calcium and phosphate ions, creating a state of supersaturation that favors remineralization through hydroxyapatite (HA) precipitation, which occludes dentinal tubules. The synergistic effect of fluoride enhances this process by forming fluorapatite (FA), further contributing to tubule occlusion [30]. However, CPP-ACP–based products cannot be used in individuals with milk protein allergies. GLUMA desensitizer, which contains glutaraldehyde and 2-hydroxyethyl methacrylate (HEMA), induces coagulation of serum albumin within dentinal fluid. This interaction leads to HEMA polymerization and the formation of a coagulation plug, a mechanism comparable to the melted layer produced after laser irradiation [17, 18]. This systematic review has several important limitations that must be considered when interpreting the findings. Although all included studies used comparable outcome measures to assess DH, they involved a relatively small number of participants and evaluated a limited number of teeth, which reduces the generalizability of the results. Across the 131 participants, a total of 462 teeth were analyzed, with the tooth serving as the unit of analysis. However, the handling of multiple teeth from the same participant varied among studies: one study [17] treated teeth as independent observations without accounting for within-subject clustering, whereas the other two studies [18, 19] applied Bonferroni-adjusted comparisons within their respective analytical designs. These inconsistencies in statistical handling raise concerns regarding the observations. Methodological quality among the included studies was limited, with two studies rated at high risk of bias [17, 18] and one presenting some concerns [19]. Although the randomization process, adherence to intended interventions, and completeness of outcome data were adequately reported, evaluator blinding was described in only one study [19], which is notable given that DH is a patient-reported outcome and therefore susceptible to detection bias. Additionally, all studies showed some concerns related to selective reporting. Participants ranged from 6 to 14 years old, which is an important factor because MIH-related hypersensitivity is typically more intense in younger children and tends to diminish with age as dentin matures and teeth are repeatedly exposed to remineralizing agents [31]. Additional variability in laser parameters, treatment protocols, comparator interventions, and follow-up periods, with only one study [18] assessing outcomes at six months, further limits the ability to draw robust conclusions regarding the long-term effectiveness of laser therapy for MIH-related DH. Additionally, although all studies stated that MIH was diagnosed according to EAPD criteria [20], none reported MIH severity distribution, which limits the ability to stratify outcomes by defect severity. Due a meta-analysis could not be conducted, the certainty of the evidence could not be formally assessed, further limiting the strength of the conclusions. Finally, the lack of a gold-standard laser protocol and the variation in device specifications limit comparability across studies. Practical considerations such as device cost and the need for professional training may further restrict clinical applicability. In contrast, desensitizing agents such as fluoride varnish are already widely used in routine care and may represent more accessible alternatives for managing MIH-related DH. Overall, these methodological and clinical limitations highlight the need for well-designed randomized clinical trials with standardized laser parameters, adequate statistical handling of clustered data, blinded outcome assessment, and long-term follow-up to clarify the true efficacy of laser therapy for reducing dentin hypersensitivity in MIH-affected teeth.

## Conclusion

Available evidence remains limited and heterogeneous due to few included studies and an overall fragile evidence base, indicating that laser therapy may reduce DH in MIH-affected teeth. Well-designed and standardized randomized clinical trials are still needed to strengthen the evidence base.

## Data Availability

No datasets were generated or analysed during the current study.
